# A novel candidate HIV vaccine vector based on the replication deficient *Capripoxvirus*, Lumpy skin disease virus (LSDV)

**DOI:** 10.1186/1743-422X-8-265

**Published:** 2011-05-30

**Authors:** Yen-Ju Shen, Enid Shephard, Nicola Douglass, Nicolette Johnston, Craig Adams, Carolyn Williamson, Anna-Lise Williamson

**Affiliations:** 1 Institute of Infectious Disease and Molecular Medicine, Faculty of Health Sciences, UCT, Observatory 7925, Cape Town, South Africa; 2 Division of Medical Virology, Department of Clinical Laboratory Science, Faculty of Health Sciences, UCT, Observatory 7925, Cape Town, South Africa; 3 Department of Medicine, Faculty of Health Sciences, UCT, Observatory 7925, Cape Town, South Africa; 4 National Health Laboratory Service, Observatory 7925, Cape Town, South Africa

## Abstract

**Background:**

The *Capripoxvirus*, Lumpy skin disease virus (LSDV) has a restricted host-range and is being investigated as a novel HIV-1 vaccine vector. LSDV does not complete its replication cycle in non-ruminant hosts.

**Methods:**

The safety of LSDV was tested at doses of 10^4 ^and 10^6 ^plaque forming units in two strains of immunocompromised mice, namely RAG mice and CD4 T cell knockout mice. LSDV expressing HIV-1 subtype C Gag, reverse transcriptase (RT), Tat and Nef as a polyprotein (Grttn), (rLSDV-grttn), was constructed. The immunogenicity of rLSDV-grttn was tested in homologous prime-boost regimens as well as heterologous prime-boost regimes in combination with a DNA vaccine (pVRC-grttn) or modified vaccinia Ankara vaccine (rMVA-grttn) both expressing Grttn.

**Results:**

Safety was demonstrated in two strains of immunocompromised mice.

In the immunogenicity experiments mice developed high magnitudes of HIV-specific cells producing IFN-gamma and IL-2. A comparison of rLSDV-grttn and rMVA-grttn to boost a DNA vaccine (pVRC-grttn) indicated a DNA prime and rLSDV-grttn boost induced a 2 fold (p < 0.01) lower cumulative frequency of Gag- and RT-specific IFN-γ CD8 and CD4 cells than a boost with rMVA-grttn. However, the HIV-specific cells induced by the DNA vaccine prime rLSDV-grttn boost produced greater than 3 fold (p < 0.01) more IFN- gamma than the HIV-specific cells induced by the DNA vaccine prime rMVA-grttn boost. A boost of HIV-specific CD4 cells producing IL-2 was only achieved with the DNA vaccine prime and rLSDV-grttn boost. Heterologous prime-boost combinations of rLSDV-grttn and rMVA-grttn induced similar cumulative frequencies of IFN- gamma producing Gag- and RT-specific CD8 and CD4 cells. A significant difference (p < 0.01) between the regimens was the higher capacity (2.1 fold) of Gag-and RT-specific CD4 cells to produce IFN-γ with a rMVA-grttn prime - rLSDV-grttn boost. This regimen also induced a 1.5 fold higher (p < 0.05) frequency of Gag- and RT-specific CD4 cells producing IL-2.

**Conclusions:**

LSDV was demonstrated to be non-pathogenic in immunocompromised mice. The rLSDV-grttn vaccine was immunogenic in mice particularly in prime-boost regimens. The data suggests that this novel vaccine may be useful for enhancing, in particular, HIV-specific CD4 IFN- gamma and IL-2 responses induced by a priming vaccine.

## Background

HIV/AIDS is a major public health problem in many parts of the world and the long term control of this disease will only be obtained with an effective prophylactic vaccine [1]. The RV144 trial is the only phase 3 efficacy trial to have tested a prime - boost combination, consisting of a recombinant canary poxvirus vector vaccine (ALVAC-HIV expressed HIV-1 subtype B gag and protease as T-cell immunogens and the gp120 of CRF01_AE strain) with a recombinant glycoprotein 120 subunit vaccine (AIDSVAX B/E) as the boost. This trial is the only one to demonstrate some protection from HIV infection [2]. The other two efficacy trials tested homologous prime-boost regimens (gp120 protein [3] or a combination of adenovirus based vaccines [4]) and showed no protection from HIV infection. These results confirm that HIV candidate vaccines induce more effective immune responses when used in heterologous prime - boost regimens [5,6].

Homologous prime-boost vaccination regimens are not as effective in inducing strong immune responses partially due to the blunting effect of anti-vector immunity [7,8]. There is some evidence that pre-immunity to the poxvirus vectors results in lower immune responses. For example, an attenuated vaccinia virus (VV) vector expressing Japanese encephalitis virus (JEV) proteins (NYVAC-JEV), induced neutralizing antibody responses only in vaccinia-nonimmune recipients while vaccinia-immune volunteers failed to develop protective JEV antibodies. Another study with an HIV vaccine with three DNA vaccine primes and an MVA boost demonstrated that while pre-immunity to VV did not abolish the immune response the magnitude of response was lower than when there was no pre-immunity [9]. A recent HIV vaccine phase 1 clinical trial investigated up to five immunisations of MVA-HIV and fowlpox (FPV-HIV) in homologous as well as heterologous prime-boost vaccination regimens. It was demonstrated that FPV-HIV alone was poorly immunogenic and that MVA-HIV alone induced maximal responses after two immunisations. T cell reponses were significantly boosted in participants receiving the MVA-HIV priming vaccination followed by the heterologous FPV-HIV booster vaccination [10]. Prime-boost vaccination regimens are also favourable as the memory T cells induced by the secondary vaccination retain the effector memory (TEM) phenotype longer than T cells generated by priming alone [11]. Poxvirus vectors are particularly good in combination with other vaccine vectors [12]. Therefore, there is a need for the identification of additional, antigenically distinct, nonpathogenic poxviruses which could be used as vaccine vectors. These vectors could be used in conjunction with other vectors to boost immune responses to specific insert antigens, or in the construction of vaccines against new diseases.

Although many vaccine vectors have been tested in clinical trials and animal models there is no one ideal vaccine vector that could be used to simultaneously protect against various diseases. Therefore, there is a need for the discovery of novel vectors and the development of improved vectors [13,14]. Many different research groups are using the same vaccine vectors for many different antigens: however, the same vectors cannot be used as vaccine vehicles for different diseases ad infinitum due to the anti-vector immunity. As they are already in clinical trials, it is likely that poxvirus vaccine vectors may be successful in HIV, tuberculosis [15] or malaria prevention [16]. It will then depend on which field gets efficacy data first as to which disease will get first use of the vaccine vector. It is likely that this will limit the use of that vector for other diseases. The number of poxvirus vectors being developed for clinical use is limited to modified vaccinia Ankara (MVA) and the avipoxviruses, fowlpox virus and canarypox virus, as vectors for both prophylactic infectious disease vaccines and cancer treatment [17-25].

Lumpy skin disease virus (LSDV), a capripoxvirus, is the causal agent of lumpy skin disease (LSD) in cattle [26]. The Neethling strain, which was attenuated in 1968 [27] is used extensively as a prophylactic vaccine against LSD [27,28]. Recombinant LSDV is an effective vaccine vector for veterinary diseases including rabies, rift valley fever and heartwater [29-31]. LSDV does not complete its replication cycle in non-ruminant hosts [32] and therefore the attenuated Neethling strain is an excellent candidate as a live replication-deficient vaccine vector. We propose that LSDV could be used in humans as a safe replication-deficient vaccine vector, in combination with a heterologous vector, such as MVA, in the prevention of HIV-1.

## Methods

### Safety of LSDV in Immunocompromised Mice

All mouse procedures were approved by the University of Cape Town Animal Research Ethics Committee. To test the safety of wild-type LSDV, RAG mice (lacking both T-cells and B-cells) and CD4 T cell knockout mice (6-8 weeks old, 5 per group) were inoculated with 10^4 ^and 10^6 ^focus forming units (ffu) per mouse by the intramuscular route, with 50 μl injected into each quadricep muscle. Mouse weights and welfare with respect to weight, appetite, coat condition and behaviour were recorded daily for 4 weeks.

### DNA vaccine, viruses and cells

The plasmid pVRC-grttnC (manufactured by Aldevron, Fargo, ND, USA) expresses an HIV-1 subtype C polyprotein consisting of Gag, RT, Tat and Nef (grttn) and is a second generation DNA vaccine. It differs from the first generation vaccine pTHgrttnC [33] in that the pTH vector backbone has been replaced with the pVRC backbone provided by the Vaccine Research Center of the National Institutes of Health, Bethesda, Maryland, USA [34]. The HIV-1 genes (*grttn*) were modified for safety issues, codon optimized for human expression [33] and cloned downstream of the cytomegalovirus AD169 immediate-early promoter, with an enhancer intron A and a Kozak sequence.

LSDV Neethling strain, a bovine vaccine, was provided by Onderstepoort Veterinary Institute, South Africa. MVA was obtained from Dr B. Moss (NIH, USA).

Foetal bovine testes cells (FBT) were isolated from trypsinized foetal bovine testes [35] obtained from the local abattoir. Madin-Darby bovine kidney epithelial (MDBK) cells and Syrian baby hamster kidney (BHK-21) cells were obtained from the American Type Culture Collection (ATCC). Chick embryo fibroblast (CEF) cells were isolated from 10-day old embryonated chicken eggs and were maintained in 2% FCS and DMEM (Gibco™).

### Construction of transfer vectors for cloning into LSDV and MVA

Plasmid pLW-51, (kindly provided by Linda Wyatt; NIH, USA), was used as a backbone for the transfer vectors. The HIV-1 genes were derived from recently transmitted HIV-1 subtype C strains in South Africa [36]. For both the LSDV and MVA transfer vectors *grttn *was cloned directly downstream of the vaccinia virus early/late mH5 promoter. ***pLW-51grttn: ****Grttn *was excised from pJH-01 [37] using *Sal*I followed by *XmaC*I and cloned into the *Sal*I and *XmaC*I sites of pLW-51 to generate pLW-51grttn. ***pYS-05: ***MVA flanking sequences in pLW-51 were replaced with sequences from the non-essential ribonucleotide reductase gene of LSDV [38]. LSDV flank I (LF1) and LSDV flank II (LFII), DNA fragments of 508 bp and 552 bp respectively were PCR amplified from LSDV DNA using the following primer pairs:

LF1 forward: 5'-GAATTCATGGTATAAAATAAAATGGAACC-3';

LF1 reverse: 5'-GCGCGCCAAACGCTATTAATCGTTCTC-3';

LF2 forward: 5'-CTGCAGTTGAGGGAATATTCTTTTCCGG-3' and

LF2 reverse: 5'-AAGCTTGGTTATTCAAGATAATTAACAAGAG-3'.

Restriction enzyme sites *Eco*RI and *Bss*HII were included at the beginning and end of LFI respectively; *Pst*I and *Hin*dIII were added at the beginning and end of LFII respectively. Amplified fragments were cloned into the *Sma*I site of pUC19 plasmid using a Rapid DNA ligation kit (Roche, Germany). MVA flank I was excised from pLW-51 with *Eco*RI and *Bss*HII (Roche, Germany) and replaced with LFI excised from pUC19 with the same restriction enzymes. MVA flank II was then replaced with LFII using *Pst*I and *Hin*dIII to generate plasmid pYS-03. *Grttn *[39] with an upstream Kozak sequence was subcloned from pJH-01 [37] into the *Sal*I and *Xma*CI sites of pYS-03. The *E.coli *xanthine-guanine phosphoribosyl transferase gene (Gpt) under the control of the VV p7.5 promoter was excised from pGpt07/14 [40] on an *Eco*RI fragment, blunt-ended and cloned into the blunt-ended *Xho*I restriction enzyme site.

### Construction of rMVA-grttn

Chick embryo fibroblast (CEF) cells were infected with wt MVA (NIH) (0.1 pfu/cell) and transfected with 500 ng pLW-51-Grttn linearized with *Nde*I. A lysate was prepared 3 days later and used to infect BHK-21 cells. Nine rounds of plaque picking were performed. The final virus isolate expressed Grttn (indicated by positive immunostaining with an anti-RT antibody(ARP428) from the National Institute for Biological Standards and Control (NIBSC) AIDS reagent program, United Kingdom) and was negative for GUS staining. rMVA-grttn was expanded in eggs (Westwood *et al.*, 1957) and the titre determined in BHK-21 cells. MVA was detected with a rabbit anti-vaccinia antibody (Biogenesis Ltd, United Kingdom) and swine anti-rabbit HRP (Dako, Denmark). Grttn expression was detected with sheep anti-RT (ARP428) and rabbit anti-sheep HRP (Dako, Denmark). Peroxidase was reacted with o-dianisidine (Sigma-Aldrich, USA) in the presence of H_2_O_2 _to visualize infected cells. Identical virus titres were obtained irrespective of the antibody used to detect virus-infected cells, indicating all MVA-infected cells expressed Grttn.

### Preparation of rLSDV-grttn

FBT cells were infected with LSDV (0.1 ffu per cell) followed by transfection with pYS-05 (400 ng). Twenty-four hours later the medium was replaced with selection medium (25 ug mycophenolic acid (MPA) (Sigma-Aldrich, USA) and 250 ug xanthine (Sigma-Aldrich, USA) per ml of 4% FCS in DMEM/Hams-F12 (Gibco, USA)). The cells were cultured for 72 hours after which a lysate was prepared for FBT cell infection and further culture (72 hours) in selection medium for 3 passages. Serial 10-fold dilutions of the final cell lysate were used to infect 6-well plates of confluent FBT cells and blue foci, detected by standard Gus staining, were picked 72h later. Virus was released by freeze/thawing and the process of infection and purification by focus picking was performed eight times. A stock of rLSDV-grttn was prepared from infected FBT cells, which were lysed 3 days p.i. Following a low-speed centrifugation step to pellet cell debris, the virus was pelleted through a 36% sucrose cushion and resuspended in PBS. MDBK cells were used to determine the virus titre by staining with sheep anti-RT (ARP428) and rabbit anti-sheep HRP (Dako, Denmark). Infected cells were visualized by adding o-Dianisidine (Sigma-Aldrich, USA) and hydrogen peroxide (Sigma-Aldrich, USA).

### PCR analysis

PCR was used to confirm the absence of wild-type LSDV in the rLSDV-grttn preparation. Three primers were designed: SQRR1, 5'-GTGGGCGTCAATGTTGAC-3' binds specifically to a sequence immediately upstream of the ribonucleotide reductase gene (insertion site). SQGrttn3, 5'-GCTACTTCCCCGACTGGC-3' binds specifically to sequences within and towards the 3' end of the Grttn gene. Anti-sense primer SQRR2, 5'-CATAAAATCAGTACATGCATCC-3' binds specifically to a sequence immediately downstream of the ribonucleotide reductase gene. Confluent monolayers of FBT cells were infected with rLSDV-grttn or wild-type LSDV at a m.o.i of 0.05 pfu per cell. 48 hours post infection lysates were prepared as described by [41]. PCR was performed, using Pfu polymerase (Promega, USA), with an initial denaturation step of 95°C for 2 minutes, followed by 35 cycles of 95°C for 1 minute, 55°C for 30 seconds and 73°C for 5 minutes; the final elongation step was at 73°C for 7 minutes.

### Immunofluorescence

FBT cells, grown on glass cover slips, were infected with LSDV and/or transfected with pYS-05, and fixed after 24 hours with methanol-acetone (1:1). Grttn protein was detected with sheep anti-p24 (Aalto Bioreagents Ltd., Ireland) and FITC conjugated donkey anti-sheep (Dako, Denmark) and viewed using a Zeiss fluorescence microscope.

### Western blot Analysis to detect protein expression

Cell lysates were prepared 24 h after infection of FBT cells with wild type LSDV (wt LSDV)or LSDVgrttn. For detection of transient expression some wells were transfected with pYS-05 follwing infection with wtLSDV. Proteins were separated using 12% SDS polyacrylamide gel electrophoreses and transferred to a nitrocellulose membrane (Amersham Hybond™-P) using a semi-dry blotting apparatus (Bio-Rad Laboratories, USA). Grttn protein was detected by probing with sheep anti-RT (ARP428) followed by anti-sheep IgG antibody conjugated with alkaline phosphatase and visualized with NBT/BCIP (Roche, Germany).

### Mouse immunizations

BALB/c mice (5 mice per group) were used for immune response evaluation. Wild type LSDV (10^6 ^ffu or 10^4 ^ffu in 100 μl PBS), rLSDV-grttn (10^6 ^ffu in 100 μl PBS), rMVA-grttn (10^6 ^plaque forming units (pfu) in 100 μl PBS) or pVRC-grttn (100 μg DNA in 100 μl PBS), was administered by the intramuscular route, with 50 μl injected into each quadricep muscle according to approval of the UCT Animal Research Ethics Committee.

### IFN-γ and IL-2 ELISPOT assays

IFN-γ and IL-2 Gag- and RT- specific CD8+ and CD4+ T cells were detected using ELISPOT sets (BD Pharmingen) and splenocytes (pooled from 5 mice per group) after red blood cell lysis [42,43]. Triplicate reactions contained splenocytes (500 000 per well) in 200 μl R10 culture medium (RPMI with 10% heat inactivated FCS, (Gibco, USA) containing 15 mM β-mercaptoethanol, 100 U penicillin per ml, and 100 μg streptomycin). Peptides (>95% pure; Bachem, Switzerland) AMQMLKDTI (GagCD8), NPPIPVGRIYKRWIILGLNK (GagCD4), VYYDPSKDLIA (RTCD8) and PKVKQWPLTEVKIKALTAI (RTCD4) were used at 4 μg/ml. Reactions without peptide were included to determine background responses. Peptide responses are reported as spot-forming units (sfu) per 10^6 ^splenocytes after subtraction of background responses. Cumulative ELISPOT responses to Gag and RT were calculated as the sum of responses to the individual CD8+ and CD4+ T cell peptides.

### Quantification of antigen-specific cytokine production

Splenocytes (7.5 × 10^6 ^per ml R10) were cultured for 24 hours with Gag and RT CD8 and CD4 peptides as used in the IFN-γ ELISPOT assay. IFN-γ in the culture supernatant was quantified using a flow cytometric bead based assay (BD Pharmingen) and calculated as pg IFN-γ per 10^6 ^splenocytes [43]. IFN-γ produced during stimulation with the individual CD8+ and CD4+ T cell peptides is expressed as cumulative IFN-γ produced.

### Pentamer staining

Splenocytes (5 × 10^6^) were labeled for 30 min at 4°C with APC-conjugated pentameric H-2D^k ^AMQMLKDTI or H-2D^d ^VYYDPSKDLIA complexes (ProImmune, Oxford) and PerCP-conjugated anti-CD8α (clone 53-6.7, eBiosciences) [43]. Flow cytometry (FACScalibur with CellQuest software (BD Biosciences)) was used to analyze pentameric-positive cells as a percentage of gated CD8+ T cells.

### Statistical analysis

Statistical tests were performed using 2-sample Student's t-test and one-way ANOVA test and p values of <0.05 were considered significant.

## Results

### Response of immunocompromised mice to LSDV

The weight fluctuations of RAG mice and CD4 T cell knockout mice over 4 weeks after vaccination with 10^4 ^ffu or 10^6 ^ffu of wild-type LSDV was no different from that of uninoculated mice or mice inoculated with PBS. No changes in appetite, coat condition and behaviour could be detected during the observation period after vaccination of these mice with wild-type LSDV.

### Construction of rLSDV-grttn and rMVA-grttn

**Figure 1:**
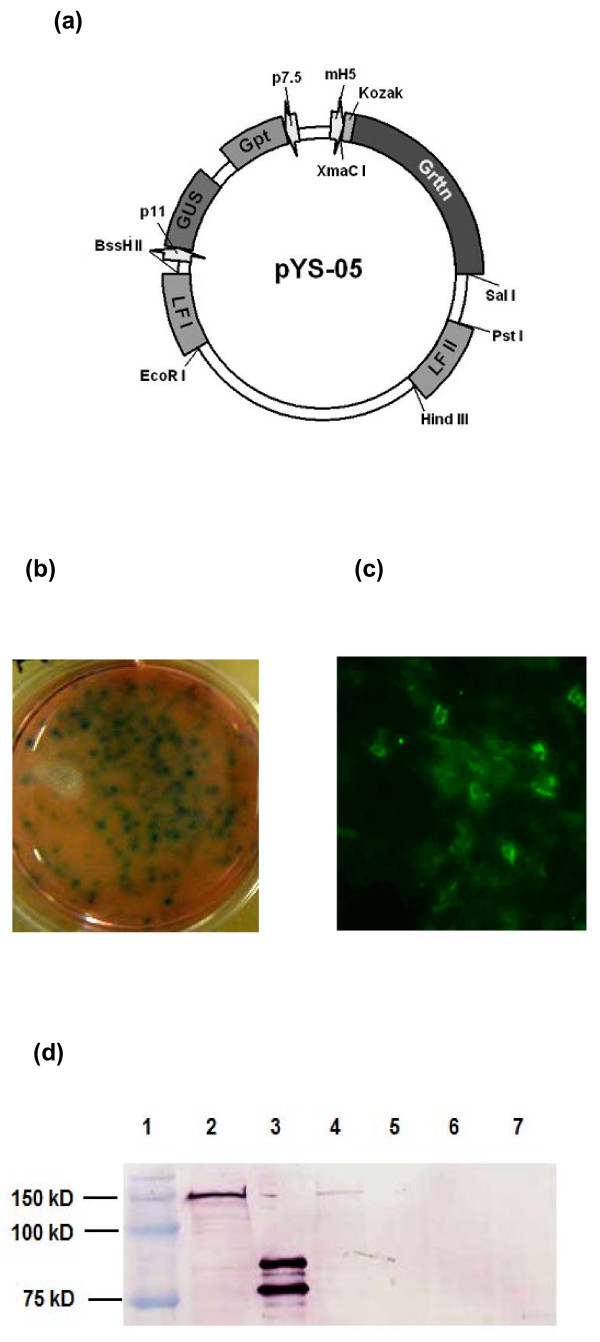
**Construction of rLSDV-grttn**. (a) transfer vector pYS-05. LSDV infection (0.1 ffu/cell) and pYS-05 transfection (100 ng DNA) of FBT cells and detection 24 hours later of (b) GUS using standard staining procedures and (c) Grttn in the cytosol of the cells by immunofluorescence using sheep anti-p24 and FITC conjugated anti-sheep. (d) Western blot detection of Grttn in cell lysates using sheep anti-RT and alkaline phosphatase conjugated anti-sheep. Lane 1, Molecular weight markers; Lane 2, pTHgrttnC (DNA) transfected cells (positive control); Lane 3, RT positive control protein standard; Lane 4, LSDV infected and pYS-05 transfected FBT cell lysate; Lane 5, LSDV-infected FBT cells; Lane 6, pYS-05-transfected FBT cells; Lane 7, uninfected FBT cells.

**Figure 2:**
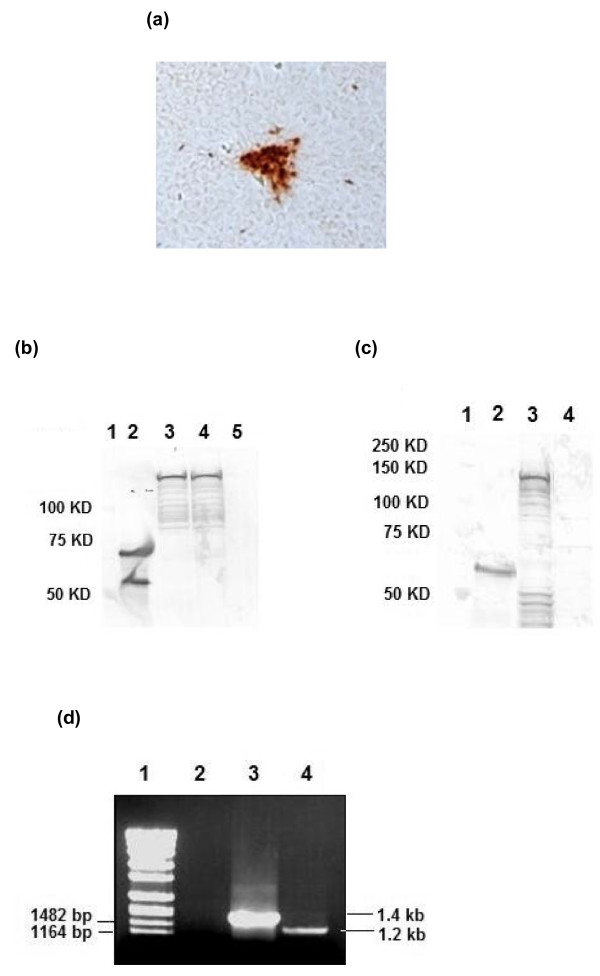
**Expression of Grttn in rLSDV-grttn infected FBT cells**. (a) Immunostain of rLSDV-grttn-infected FBT cells incubated with RT specific sheep antibody, followed by peroxidase conjugated anti-sheep immunoglobulin antibody. (100X magnification). (b) Western blot analysis of cell lysates using sheep anti-RT and alkaline phosphatase conjugated anti-sheep. Lane 1, molecular weight markers; Lane 2, RT positive control protein standard; Lane 3, rMVA-grttn infected BHK-21 cells; Lane 4, rLSDV-grttn-infected FBT cells; Lane 5, LSDV infected FBT cells (c) Western blot analysis of cell lysates using sheep anti-p24 and alkaline phosphatase conjugated anti-sheep. Lane 1, molecular weight markers; Lane 2, Gag positive control protein standard; Lane 3, rLSDV-grttn infected FBT cells; Lane 4, LSDV infected FBT cells. (d) PCR analysis of rLSDV-grttn using primers designed to amplify a 1.4 kb fragment from the wild type LSDV and 1.2 kb fragment from the recombinant rLSDV-grttn. DNA products were subjected to 1% agarose gel electrophoresis. Lane 1, DNA molecular weight standards with relevant sizes indicated to the left; Lane 2, lysates of FBT cells; Lane 3, LSDV-infected FBT cells; Lane 4, rLSDV-grttn infected FBT cells.

Recombinant LSDV and MVA expressing Grttn, the HIV-1 subtype C polyprotein comprising Gag, RT, Tat and Nef [44], were constructed. Initially, transient expression assays were performed to test for gene expression from vaccinia virus promoters in LSDV-infected FBT cells which were transfected with pYS-05. Beta-glucuronidase (GUS) expression was detected 24 hours post infection of foetal bovine testes (FBT) cells with LSDV and transfection with pYS-05 (Figure 1b). No expression was observed in negative controls (uninfected cells; LSDV infected, non-transfected cells; uninfected, pYS-05 transfected cells). Transient expression of Grttn (150kDa) using an anti-p24 antibody was observed by immunofluorescence 24 hours post LSDV infection and pYS-05 transfection of FBT cells (Figure 1c). and confirmed by western blot analysis (Figure 1d, lane 4). After further passaging a stable recombinant rLSDV-grttn was obtained and titrated in Madin-Darby bovine kidney (MDBK) cells. Foci of infection were detected by immunostaining for RT expression (Figure 2a). Expression of full length Grttn (150 kDa) was confirmed by western blot analysis of lysates from virus-infected FBT cells (Figure 2b, lane 4 (RT) and Figure 2c, lane 3 (Gag)). No Grttn expression was detected in wild type LSDV infected cells (lanes 5 and 4 in Figures 2b and 2c respectively). The absence of wild type LSDV in the purified rLSDV-grttn stock was confirmed by PCR (Figure 2d). The expected PCR DNA fragment sizes of 1.2 kb and 1.4 kb were amplified from recombinant and wild type virus lysates respectively. The absence of a 1.4 kb fragment in rLSDV-grttn-infected cells (Figure 2d, lane 4) confirmed the absence of any residual wild type virus present in the rLSDV-grttn stock.

### rLSDV is immunogenic and boosts a primary DNA vaccination

The immunogenicity of rLSDV-grttn was evaluated in BALB/c mice using rLSDV-grttn alone as well as after priming with the DNA vaccine pVRC-grttnC, which expresses Grttn. Comparative experiments were performed using rMVA-grttn and pVRC-grttnC.

**Figure 3:**
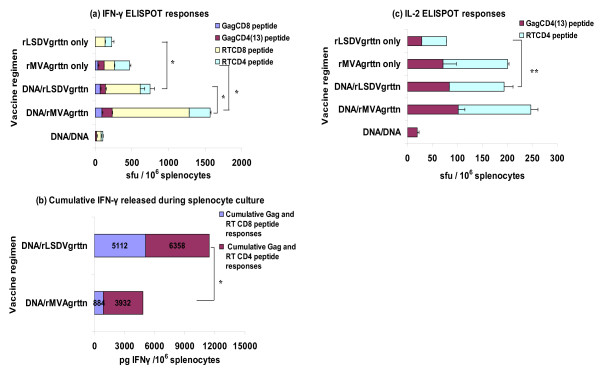
**HIV-specific T cell responses to a DNA vaccine prime and boost with rLSDV-grttn or rMVA-grttn**. Groups of mice were vaccinated with the DNA vaccine pVRC-grttn then boosted on day 28 with the DNA vaccine, rLSDV-grttn or rMVA-grttn. Two other groups of mice were vaccinated on day 28 with rLSDV-grttn or rMVA-grttn. Mice in all the groups were killed on day 40, 12 days after the final immunization. Splenocytes, pooled from 5 mice per group, were used in the immunological assays. (a) IFN-γ ELISPOT assay with Gag and RT CD8 and CD4 peptides. Bars are the mean ± the SD of spot forming unit (sfu) from triplicate reactions for 10^6 ^splenocytes after subtraction of background spots in the absence of peptide. (b), splenocytes were also cultured with the individual HIV CD8+ and CD4+ T cell peptides for 48 h and culture supernatants were collected and IFN-γ (pg cytokine/10^6 ^splenocytes) content quantified. The sum of IFN-γ produced during stimulation with the individual CD8+ and CD4+ T cell peptides was calculated and is presented as the cumulative total IFN-γ produced by HIV-specific CD8+ T cells or CD4+ T cells, values are indicated on the bars; and (c) IL-2 ELISPOT assay with pooled splenocytes and peptides as used in the IFN-γ ELISPOT assay. *: p < 0.01; **: p < 0.05. Data are from a representative experiment.

A single vaccination with rLSDV-grttn induced a cumulative IFN-γ ELISPOT response of 224 sfu/10^6 ^splenocytes to the RT CD8 and CD4 peptides with no response to Gag. In comparison a single inoculation with rMVA-grttn induced cumulative responses to Gag and RT CD8 and CD4 peptides of 467 sfu/10^6 ^splenocytes (Figure 3a). Both rLSDV-grttn and rMVA-grttn boosted the immune response induced by a pVRC-grttn prime. A prime with pVRC-grttn and boost with rLSDV-grttn induced a cumulative Gag and RT CD8 and CD4 IFN-γ ELISPOT response of 747 sfu/10^6 ^splenocytes with 540 sfu/10^6 ^splenocytes (72%) from CD8 cells. A significantly greater response (p < 0.01) of 1567 sfu/10^6 ^splenocytes with 1139 sfu/10^6 ^splenocytes from CD8 cells (72%) was achieved with a rMVA-grttn boost (Figuer 3a). These responses were considerably greater (p < 0.01) than the cumulative response of 107 sfu/10^6 ^splenocytes induced by two pVRC-grttn vaccinations (Figure 3a).

The magnitude of IFN-γ released from splenocytes during culture with Gag and RT peptides was measured. A pVRC-grttn prime and rLSDV-grttn boost produced a total of 11470 pg IFN-γ/10^6 ^splenocytes of which 45% (5112 pg IFN-γ/10^6 ^splenocytes) was from CD8 Gag- and RT-specific cells. This is significantly (p < 0.01) higher that a rMVA-grttn boost which induced a total of 4816 pg IFN-γ/10^6 ^splenocytes with 19% (884 pg IFN-γ/10^6 ^splenocytes) from CD8 Gag and RT-specific cells (Figure 3b). The cumulative frequency of Gag- and RT-specific CD4 and CD8 cells counted in the IFN-γ ELISPOT assay was collated with the level of IFN-γ released by these cells in culture as a further comparison of the regimens. Gag- and RT-specific CD8 and CD4 cells induced by a DNA prime and rLSDV-grttn boost were calculated to produce 9 and 31 pg IFN-γ/splenocyte respectively while these cells induced by a DNA prime and rMVA-grttn boost were calculated to produce 1 and 10 pg IFN-γ/splenocyte respectively (Figure 3a and Figure 3b).

HIV-specific CD4 cells producing IL-2 were generated by the individual poxvirus vaccines and by the DNA vaccine prime and rLSDV-grttn or rMVA-grttn boost vaccination regimens (Figure 3c). Only the DNA prime/rLSDV-grttn boost regimen resulted in the cumulative response to Gag and RT CD4 peptides (194 sfu/10^6 ^splenocytes) being greater than the sum of the cumulative Gag and RT CD4 peptide responses for the individual vaccines (p < 0.05) indicating a true boost of CD4 IL-2 producing cells by the DNA/rLSDV-grttn vaccine regimen (Figure 3c).

### Responses to heterologous combinations of rLSDV-grttn and rMVA-grttn

**Figure 4:**
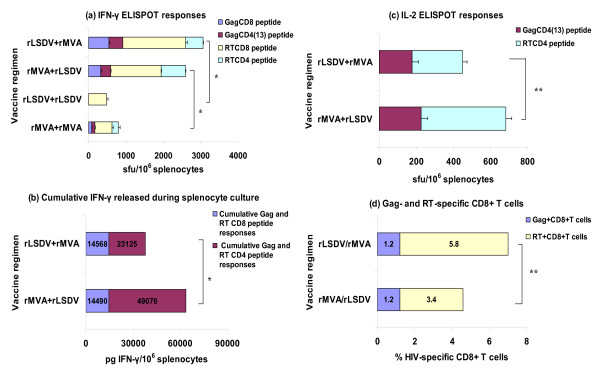
**HIV-specific T cell responses to poxvirus vaccine prime and boost vaccination regimens**. Groups of mice were primed with rLSDV-grttn or rMVA-grttn then boosted on day 28 with either rMVA-grttn or rLSDV-grttn as indicated on the y-axis. Mice in all the groups were killed on day 40, 12 days after the final immunization. Splenocytes pooled from 5 mice per group were used in the immunological assays. (a) IFN-γ ELISPOT assay with Gag and RT CD8 and CD4 peptides. Bars are the mean ± the SD of spot forming unit (sfu) from triplicate reactions for 10^6 ^splenocytes after subtraction of background spots in the absence of peptide. (b), splenocytes were also cultured with the individual HIV CD8+ and CD4+ T cell peptides for 48 h and culture supernatants were collected and IFN-γ (pg cytokine/10^6 ^splenocytes) content quantified. The sum of IFN-γ produced during stimulation with the individual CD8+ and CD4+ T cell peptides was calculated and is presented as the cumulative total IFN-γ produced by HIV-specific CD8+ T cells or CD4+ T cells, values are indicated on the bars; and (c) IL-2 ELISPOT assay with pooled splenocytes and peptides as used in the IFN-γ ELISPOT assay; (d) pooled splenocytes were also labelled with APC-conjugated H-2D^k ^pentameric complexes folded with the Gag AMQMLKDTI peptide or the RT peptide VYYDPSKDLIA and PerCP-conjugated anti-CD8α on day 28. Gag and RT -specific MHC class I pentameric complex binding is expressed as a percentage of gated CD8+ T cells, percentages are indicated on the bars. *: p < 0.01; **: p < 0.05. All data are from a representative experiment.

The potential use of rLSDV-grttn in heterologous poxvirus prime and boost vaccination regimens with rMVA-grttn was investigated (Figure 4). A cumulative Gag and RT CD8 and CD4 response of 2628 sfu/10^6 ^splenocytes with 1000 sfu/10^6 ^splenocytes (64%) from responding CD8+ T cells was measured in the IFN-γ ELISPOT assay for a prime with rMVA-grttn and a boost with rLSDV-grttn (Figure 4a). This response was significantly greater (p < 0.01) than the cumulative response of 795 sfu/10^6 ^splenocytes (68% CD8) achieved with a prime and boost with rMVA-grttn (Figure 4a). The magnitude of a rLSDV-grttn prime and rMVA-grttn boost was similar to the reverse prime-boost with a cumulative Gag and RT CD8 and CD4 response of 3066 sfu/10^6 ^splenocytes in the IFN-γ ELISPOT assay where 2214 sfu/10^6 ^splenocytes (72%) were from responding CD8+ T cells. This was significantly (p < 0.01) higher than a prime and boost with rLSDV-grttn which generated 477 sfu/10^6 ^splenocytes from only RT CD8 cells (Figure 4a).

The magnitude of IFN-γ produced by Gag- and RT- specific cells during culture was quantified for the heterologous poxvirus prime-boost vaccination regimens (Figure 4b). A total of 63566 pg IFN-γ/10^6 ^splenocytes (with 14490 pg IFN-γ/10^6 ^splenocytes (23%) from CD8 cells) was produced by a rMVA-grttn prime and rLSDV-grttn boost (Figure 4b). A significantly (p < 0.01) lower level of IFN-γ (total of 37693 pg IFN-γ/10^6 ^splenocytes with 14568 pg IFN-γ/10^6 ^splenocytes (39%) from Gag and RT CD8 cells) was induced by a rLSDV-grttn prime and rMVA-grttn boost. Comparing the heterologous prime-boost vaccine regimens in terms of levels of cytokines produced by individual splenocytes related to frequency of responding cells in the IFN-γ ELISPOT assay, for a rMVA-grttn prime and rLSDV-grttn boost, 7 pg and 53 pg IFN-γ were produced per responding Gag- and RT-specific CD8+ and CD4+ T cell respectively. In contrast Gag and RT-specific CD8+ and CD4+ T cells for a rLSDV-grttn prime and rMVA-grttn boost produced 9 pg IFN-γ and 28 pg IFN-γ per responding cell respectively (Figure 4a and 4b). Thus the rMVA-grttn prime and rLSDV-grttn boost regimen induced HIV-specific CD4+ T cells with a higher capacity to produce IFN-γ than a rLSDV-grttn prime and rMVA-grttn boost.

Gag and RT CD4 IL-2 ELISPOT responses were detected for the heterologous prime-boost vaccination regimens (Figure 4c). The cumulative IL-2 ELISPOT response for rMVA-grttn prime and rLSDV-grttn boost reached 684 sfu/10^6 ^splenocytes. This response was 450 sfu/10^6 ^splenocytes for a rLSDV-grttn prime and rMVA-grttn boost (Figure 4c).

Binding of H-2D^k ^AMQMLKDTI or H-2D^d ^VYYDPSKDLIA complexes to CD8+ T cells indicated both heterologous poxvirus vaccination regimens induced high frequencies of HIV-specific CD8+ T cells (Figure 4d). When rLSDV-grttn was the booster vaccine the sum of Gag- and RT- specific CD8 cells reached a frequency of 4.6% of the CD8 cells while this frequency was 7% of the CD8 cells when rMVA-grttn was the booster vaccine (P < 0.05; Figure 4d). Approximately 98% of these HIV-specific CD8 cells for both vaccination regimens expressed CD44 confirming these cells to be antigen experienced.

Insight into the fraction of total HIV-specific CD8 cells in the spleen that produce IFN-γ in response to the poxvirus prime and boost regimens was obtained by comparing the pentameric H-2D peptide complex binding data with the frequency of Gag- and RT-specific CD8 cells detected in the IFN-γ ELISPOT assay. For this the cumulative frequencies of Gag- and RT-specific CD8 cells determined in the IFN-γ ELISPOT assay was expressed as a percentage of the total CD8 population (136000 CD8 cells/10^6 ^splenocytes) in the spleen. When rLSDV-grttn was the booster vaccine HIV-specific CD8 cells responding in the IFN-γ ELISPOT assay was calculated to be 1.9% of the CD8 population in the spleen. This figure was calculated to be 2.2% of the CD8 population in the spleen when rMVA-grttn was the booster vaccine. It thus appears if these figures are compared to the direct enumeration of HIV-specific CD8 cells using H-2D peptide complex binding that these vaccine regimens induce HIV-specific CD8 cells that produce more than HIV-specific IFN-γ producing cells.

## Discussion

LSDV (Neethling) has a long history of safety in animals as a veterinary vaccine. This, together with its restricted host range properties, makes it attractive as a potential safe vaccine vector for an HIV vaccine. Our experiments demonstrated LSDV safety in immunocompromised (RAG and CD4 knockout ) mice at 10^4 ^and 10^6 ^ffu per mouse. This is a similar safety profile as reported in immunodeficient mice for MVA, although it should be noted that MVA was also tested at a higher titre of 10^9 ^[45]. These results indicate that the vectors should be safe in humans at similar doses.

A potential disadvantage is that lumpy skin disease is confined to Africa and Asia, suggesting the LSDV vector may be limited to use in these two continents. However, the enormous extent of the public health problem caused by HIV in Southern Africa and the urgency with which vaccine intervention is required in this region justifies the development of this African HIV vaccine vector. Although LSDV grows optimally in primary FBT cells, these cells would probably not be approved by regulatory bodies for the mass production of an HIV-1 vaccine. An alternative approach to the large scale growth of LSDV would be to grow the virus on the chorioallantoic membranes (CAMs) of fertilized hens' eggs or primary egg cell culture. The Neethling strain of LSDV was attenuated by passage of the virus both in primary cells as well as on the CAMs of fertilized eggs [28].

Poxvirus based vaccines are known to activate both humoral and cellular immune responses [46]. The immunological correlates of protection against HIV have not been identified but there is increasing evidence from animal studies and T-cell responses to HIV that T cell participation is involved [47-49]. This is also supported by correlations between HIV specific CTL and the control of disease progression observed in certain long-term non progressing HIV-1 infected individuals [50,51]. Excellent safety features of MVA are promoting endeavours concentrating on developing new MVA constructs with improved immunogenicity [52]. The data in this study indicated rLSDV-grttn is immunogenic with a single vaccination inducing HIV specific IFN-γ and IL-2 HIV-specific CD8+ and CD4+ T cells. An important finding is the suggestion that rLSDV-grttn can be used successfully as a booster vaccine. Boosted responses were achieved after a prime with either a DNA vaccine, pVRC-grttn, or rMVA-grttn vaccine and boost with rLSDV-grttn. In this study a comparison was made between using rLSDV-grttn and rMVA-grttn as the booster vaccines. The frequency of total HIV-specific IFN-γ positive T cells in response to a DNA prime and rLSDV-grttn boost was lower than that of a DNA prime and rMVA-grttn boost regimen. When the level of IFN-γ released by the HIV-specific CD8+ and CD4+ T cells during culture with the HIV peptides and the frequency of cells responding to these peptides in the IFN-γ ELISPOT assay was compared it appears that the HIV-specific cells have a greater capacity by a factor of 2 to produce IFN-γ when rLSDV-grttn is the booster vaccine than when rMVA-grttn is the booster vaccine. A further contrast between the use of rLSDV-grttn and rMVA-grttn as the booster vaccine is the finding that the total T cell IFN-γ production is equally from CD8+ and CD4+ T cells when rLSDV-grttn is the booster vaccine but predominantly produced by CD8+ T cells when rMVA-grttn is the booster vaccine.

Although homologous boosting with rLSDV-grttn and rMVA-grttn did yield improved responses over that of a single vaccination with each of these poxvirus vaccines, a much improved response was obtained if the poxvirus vaccines were used in heterologous prime-boost vaccination regimens. Both heterologous poxvirus vaccine regimens induced high frequencies of HIV-specific CD8 cells as determined by binding of pentameric H-2D complexes folded with the Gag and RT CD8 peptides to CD8 cells in the spleen. These HIV-specific CD8+ T cells expressed CD44 confirming these cells to be antigen experienced. IFN- γ and IL-2 ELISPOT assays indicated cells producing IFN-γ or IL-2 were induced. A boost with either rLSDV-grttn or rMVA-grttn induced similar frequencies of HIV-specific CD8+ and CD4+ IFN-γ or IL-2 producing T cells. However, the more potent heterologous prime-boost inoculation regimen with respect to IFN-γ production was identified by quantifying the level of IFN-γ released by the HIV-specific T cells during culture with the HIV peptides. The HIV-specific CD8 and CD4 cells induced by a prime with rMVA-grttn and boost with rLSDV-grttn produced 1.7 fold more IFN-γ into culture supernatants than the reverse inoculation regimen. The difference between the regimens is attributable to increased IFN-γ production by HIV-specific CD4 cells by the rMVA-grttn prime and rLSDV-grttn boost regimen. Responses to rLSDV-grttn appear to be biased towards CD4 cell responses. For both heterologous poxvirus inoculation regimens the HIV-specific CD8 cells produced approximately equal levels of IFN-γ per HIV -specific CD8 cell. However the CD4 cells from the rMVA-grttn prime and rLSDV-grttn boost regimen produced almost twice more IFN-γ per HIV -specific CD4 T cell than the rLSDV-grttn prime and rMVA-grttn boost.

## Conclusions

These results indicate that LSDV (Neethling) could well be a promising novel vaccine vector for use in Africa as an HIV-1 vaccine. We have shown it to be effectively immunogenic in mice, especially as a booster vaccine.

## Competing interests

The authors declare that they have patents on the vaccine inserts (CW) and LSDV as an HIV vaccine vector (ALW, ND, YS).

## Authors' contributions

ALW is the principal investigator, initiated the study, participated in the design and supervision of the project, helped to draft the manuscript and submitted the manuscript. YS constructed the recombinant LSDV, performed the immunoassays and drafted the original manuscript. ES co-ordinated the animal work, obtained ethics approval for the study, participated in the supervision of the immunoassay experiments and participated in drafting the manuscript. ND participated in the construction of recombinant LSDV, the design of the study, the drafting of the manuscript. NJ constructed recombinant MVA. CA constructed plasmid pVRC-grttnC. CW supervised the construction of pVRC-grttnC. All authors read and approved the manuscript.
